# A Case of Progressive Parenchymatous Degeneration of the Cerebellum

**Published:** 1917-02

**Authors:** Jas. N. Brawner

**Affiliations:** Atlanta, Ga.


### A CASE OF PROGRESSIVE PARENCHYMATOUS DEGENERATION OF THE CEREBELLUM.

### Occurring in a Man Fifty-Five Years of Age, Showing Marked Muscular Inco-ordination of the Cerebellar Type in All Four Extremities.

Jas. N. Brawner, M. D., Atlanta, Ga.

Mr. B., Man, aged 60. Occupation, lawyer and banker. Mother still living at age of 87. Father died at 70 of pneumonia. Married and has two healthy children with the exception that one child is rather nervous. Patient had a brother who died of a nervous trouble, but his brother showed no disturbances in gait, so far as he remembers. He also had an aunt who died of some brain disease of a progressive nature. The patient in his boyhood was very small and did not develop until he was about 18 years of age. He then grew into a healthy man. Up to five or six years ago his general health was good. About five years ago he awoke one morning to find that on arising he had vertigo, and on walking had a tendency to fall. This persisted for several days and then disappeared, excepting the ataxia. Since then he has had no vertigo, headache nor vomiting, but his ataxia has gradually progressed and during the past six months has become very marked, he being barely able to stand alone. His wife states that his condition had been diagnosed by a physician in an adjoining city as locomotor ataxia. In all probability, however, his condition was diagnosed as cerebellar ataxia, his wife misunderstanding the terms used.

Physical Examination: There is absolutely no sensory disturbances of any kind in any part of the body. His speech is slow, scanning, and explosive, showing marked ataxia of the vocalizing muscles. The explosiveness is very marked during slight emotional excitement. His expression is mask-like, rarely blinking his eyes. His pupils are small, though they re-act to light and to accommodation. His eye grounds as reported by Dr. J. D. Thompson are normal. Nystagmus is absent. There is inability to move the eyes either up or down, though they move from side to side to some extent. Hearing is normal. He is well developed muscularly, showing absolutely no hypotonus or disturbances of muscular nutrition. Hand grips are normal. The flexors, extensors, adductors and abductors, in all four extremities, are normal in tonicity and strength. On movement there is marked ataxia in all four extremities, the ataxia being the same whether eyes are open or closed. There is no loss of the conscious sense of position.

Adiadokokinesis is present to a marked extent. He is absolutely unable to walk alone, falling about helplessly in any position. Has no tendency to rotate. Lateropulsion and posteropulsion are about equal, though the ataxia on the right half of the body is more marked than on the left. The gait is typically a cerebellar one, the patient being absolutely unable to adjust himself relative to space while in a standing position. In making voluntary movements it is necessary for them to be made very slowly, as the muscular adjustments are consciously directed. He writes slowly, just like a child learning to write, and frequently his hand in making a letter will go too far. In writing there is a very slight tremor. In other tests tremor cannot be detected. His reflexes are normal, and with the exception of an arteriosclerosis and a bilateral cerebellar ataxia in both lower and upper extremities, involving also the neck, facial, and eye muscles he gives the appearance of a perfectly healthy man. His urine is normal, blood pressure 140, spinal fluid negative to the Wasserman reaction, 2 cells per c. m., very slight increase in globu lins.

Diagnosis: Tumor of the cerebellum can probably be ruled out absolutely in this case. There has been no headache, nausea, or vomiting. The onset of the disease, however, with the attack of dizziness, which later disappeared, is probably indicative of a softening process of arterial origin. The fact that the ataxia has gradually become more marked during the past four years probably indicates a parenchymatous degeneration of the cortex of the cerebellum, which may or may not be accompanied by an arteriosclerotic process. When we take into consideration the absence of nystagmus and tremor and the slow progressive nature of the disease, I think this case worth reporting. It is also remarkable that he shows no muscular weakness nor hypotonus.

702 Grant Building.

